# Expression of Concern: Akt Regulates Drug-Induced Cell Death through Bcl-w Downregulation

**DOI:** 10.1371/journal.pone.0213701

**Published:** 2019-03-19

**Authors:** The PLOS ONE Editors

Concerns have been raised about several figures in this article [1].

- Fig 1B: Lane 1 of the Akt blot is similar to lane 2 of the β-actin blot, though with different aspect ratio.
- Fig 2A: The background in lane 2 of the Akt blot is notably different than the background in other lanes. The authors have provided the original blot image in support of this panel in S1 File; the area above and below the band in lane 2 is different in the raw blot image versus the published image.
- The β-actin blot from Fig 2A was duplicated in error as representing the β-actin blot for Fig 2D, though the aspect ratio differs between the two published figure panels. The authors have provided original blot data for β-actin results in Fig 2A and 2D in S2 and S3 Files, as well as an updated version of Fig 2 in which the β-actin panel in Fig 2D is updated.
- Fig 4C: Lanes 3, 4, 5 of the Flag-Bcl-w blot appear similar to lanes 1, 2, 3, of the HA-GSK3β blot. The authors claimed that this resulted from a figure preparation error and provided original blot images supporting Fig 4 in S4 File. The blot images provided indicated that lanes had been spliced in generating the figure panels in Fig 4A and in the Flag IP/GSK3β Western blot panel of Fig 4C. The authors provide here an updated Fig 4 in which these issues have been addressed.
- Fig 7A: The authors did not explain in the article or figure legend that they had spliced together image fragments in generating the HA and EE total extract blot panels to remove lanes between lanes 2 and 3 of the published figure. The authors provided raw blot images supporting these panels in S5 and S6 Files.

Total extract blots in Fig 4C of [1] were duplicated in error in Fig 2C of [2]. The authors clarified that the correct panels were shown in the *PLOS ONE* figure; the *Cell Death and Differentiation* article was retracted in 2017 due to concerns about this figure [3].

The authors clarified that there were some errors in the reporting of the Fig 4C experiments in [1]. First, the Results section text incorrectly reports that the experiment was conducted in HEK293 cells. As noted in the figure legend, these experiments were done in HeLa cells. Second, the original published version of Fig 4C included a panel label indicating that upper blot was generated using samples from an anti-Flag immunoprecipitation. As noted in the Results text, figure legend, and updated figure provided with this notice, the immunoprecipitation was performed using an anti-HA antibody. Third, the transfections that were performed were not clearly and correctly relayed in the Fig 4C legend; this has been addressed in the updated legend provided with this notice.

The authors have provided raw data underlying graphs reported in Figs 5A and 6B (S7–S10 Files). Please note that the final graphs for Fig 5A were derived from the combination of independent experiments; the authors have provided the original raw counts for one of these experiments. S10 File also includes an updated version of the graph shown in Fig 6B, based on re-calculations from the raw data.

The authors clarified that the raw data supporting Figs 1A, 1B, 1C, 2B, 2C, 3A, 3B, 3C, 5B, 6A, 6C are no longer available due to the amount of time that has elapsed since the results were published.

Given the extent of the concerns about the published figures in this article, the *PLOS ONE* Editors issue this Expression of Concern.

The authors disagree with this Expression of Concern.

Corrected versions of Figs 2 and 4 are provided here.

**Fig 2:**
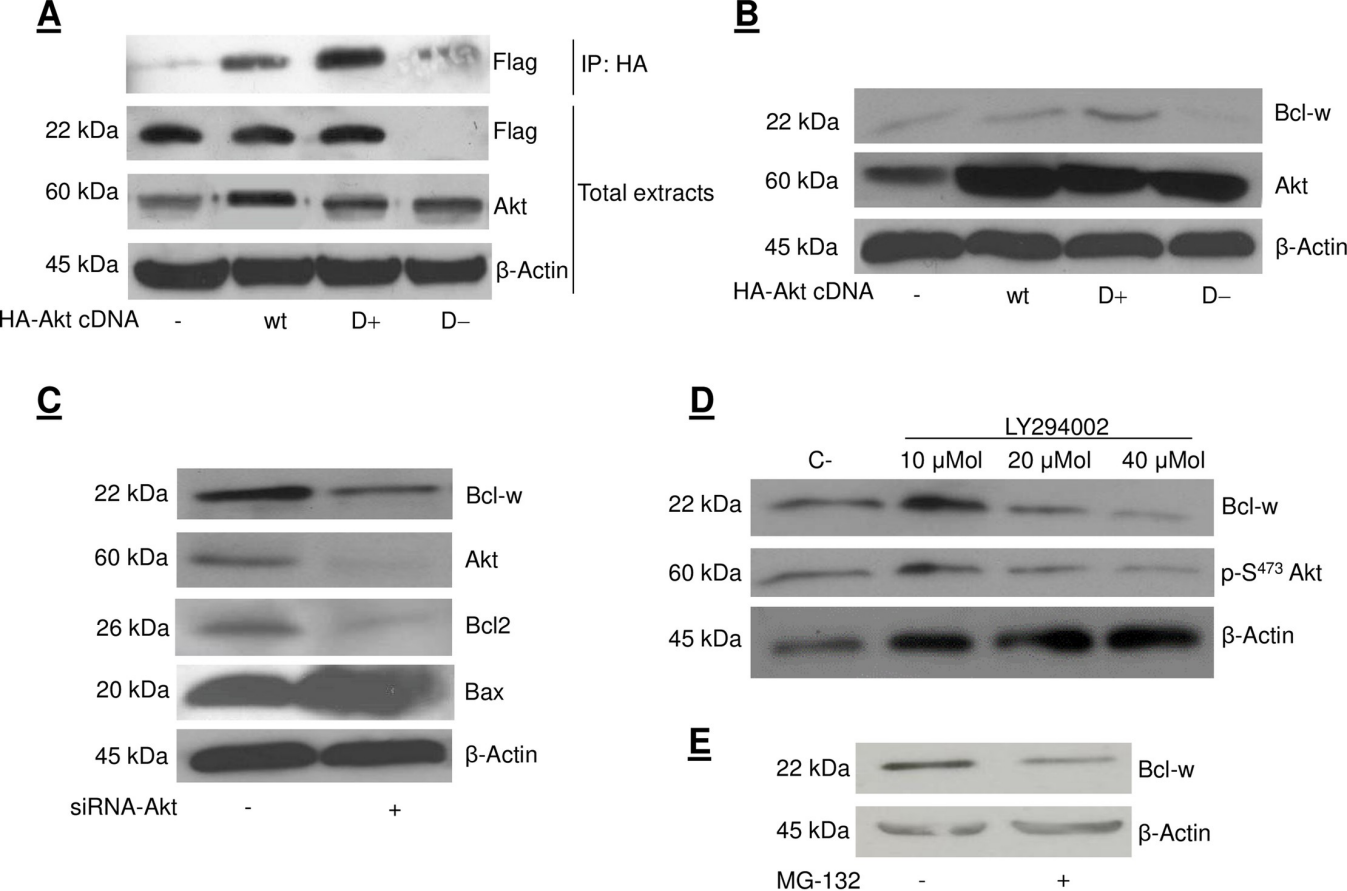
Akt activity regulates Bcl-w expression. (A) HeLa cells were transfected with 2 μg of HA-Akt wt, Akt D+, or HA-Akt D− cDNA and 2 μg Flag-Bcl-w for 48 hrs. Protein extracts were immunoprecipitated with an anti-HA monoclonal antibody. Immunoprecipitates were resolved on 12% SDS-PAGE and transferred to Hybond-C nitrocellulose. Membranes were incubated with anti-Flag antibody (0.2 μg/ml). 50 μg of total sample extracts were also analyzed by western blot using the indicated antibodies. Loading control was obtained using anti-β actin antibody. (B) HeLa cells were transfected with 4 μg of HA-Akt wt, HA-Akt D+, or HA-Akt D− cDNA for 48 hrs. Protein extracts were blotted with anti-Bcl-w antibody in order to detect endogenous levels of Bcl-w. Loading control was obtained with anti-β actin antibody. (C) Cells were transfected with 100 nM of siAkt-RNA for 48 hrs. Cellular proteins were solubilized and analyzed by western blot using the indicated antibodies. (D) HeLa cells were treated with 10, 20 or 40 μM of LY294002 for 24 hrs. Protein extracts were analyzed by western blot using the indicated antibodies. Loading control was obtained using anti-β-actin antibody. (E) Bcl-w HeLa cells were treated with 10 μM of MG-132 for 8 hrs. 40 μg of protein extracts were analyzed by western blot with anti-Bcl-w antibodies. Loading control was obtained using anti-β actin antibody.

**Fig 4:**
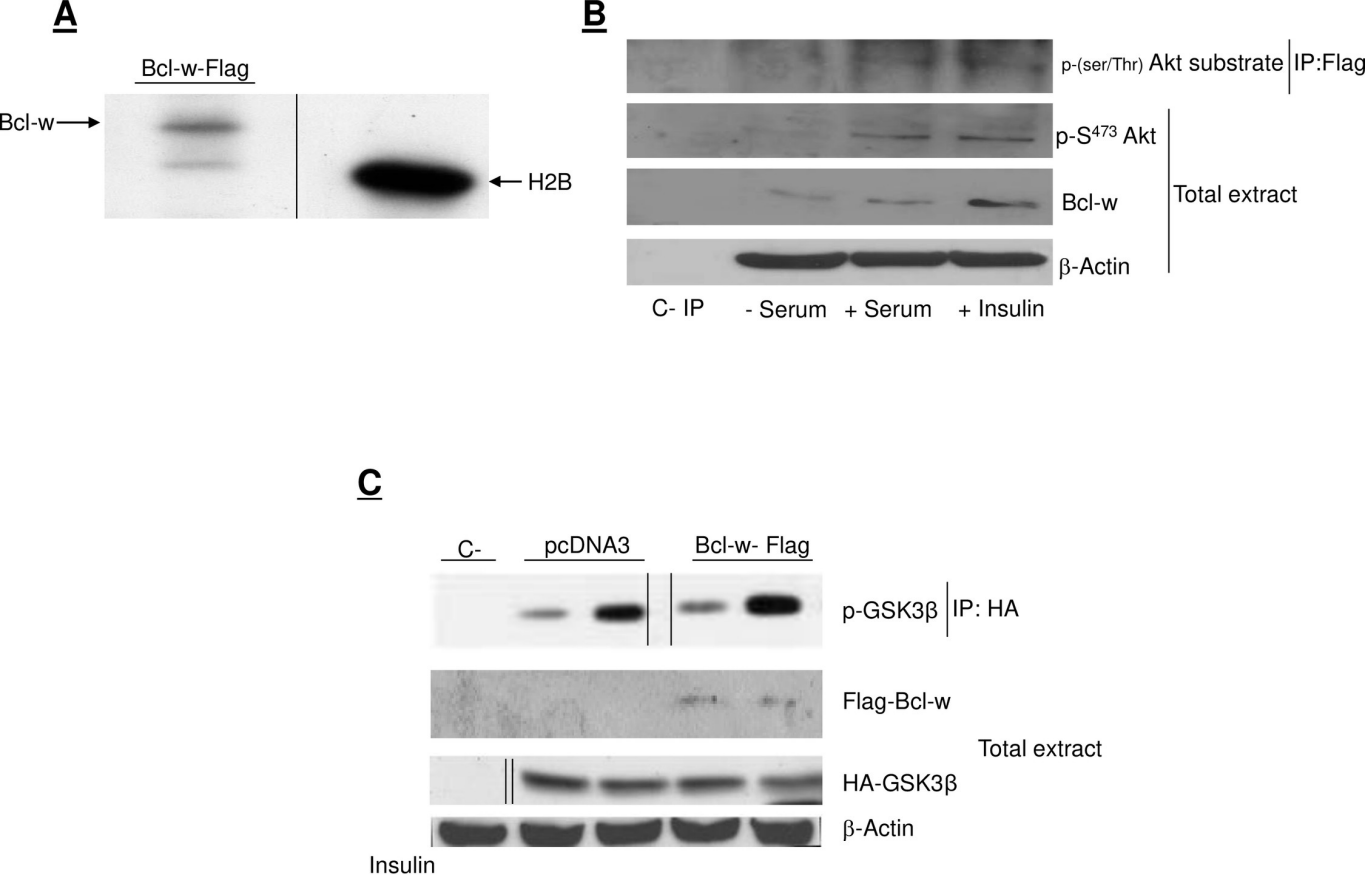
Akt phosphorylates Bcl-w in vitro and in vivo. (A) HeLa cells were transfected with 2 μg of DNA of Flag Bcl-w, solubilized, and 1 mg of protein extract was immunoprecipitated with an anti-M2 Flag antibody. Immunoprecipitates were incubated with recombinant constitutive active Akt (rAkt), and in vitro kinase assay was conducted as described in the methods. Samples were loaded onto 2.5% SDS-PAGE and analyzed by autoradiography. As positive control we used Histone2B (H2B). (B) HeLa Bcl-w stable expressing clones were serum starved for 18 hrs and then stimulated with 100 nM insulin or with 20% serum for 15 min as indicated. Cells were solubilized and immunoprecipitated with an anti-M2 Flag antibody. Immunoprecipitates were loaded onto SDS-PAGE and blotted with an anti-phospho Akt substrate antibody that recognizes all the phosphorylated Akt substrates. Total extracts were analyzed by western blot using the indicated antibodies. (C) HeLa cells were transfected with 2 μg of HA-GSK3β and either 2 μg of pcDNA3 empty vector (lane 2) or 2 μg of Flag-Bcl-w (lane 3) for 48 hrs. Cells used for the samples in lane 1 were untransfected controls (C-). Cells were stimulated with 100 nM insulin for 15 min, solubilized, immunoprecipitated using an anti-HA antibody, and analyzed by western blot using an anti-phospho-Gsk3β antibody (top panel). Total extracts were analyzed by western blot using anti-Flag, anti-HA, and anti-β-actin antibodies (panels 2–4, respectively). Bcl-w overexpression does not affect Akt activity. Vertical lines in Fig 4A and 4C indicate where fragments of the original blot images were spliced to remove or rearrange lanes. The raw uncropped blots are provided in S4 File. Note that the Flag-Bcl-w and HA-GSK3β data in the second and third panels of Fig 4C were obtained using independent blots for which the corresponding β-Actin control blots are provided in S4 File. The β-Actin panel shown in the figure is for the anti-HA blot, but the authors clarified that the same protein extracts were used for both blots.

## Supporting information

S1 File Raw blots provided in support of Akt panel in Fig 2A. (PPTX)

S2 File Raw blot supporting β-Actin results in Fig 2A. (JPG)

S3 File Correct original raw blot for β-Actin results in Fig 2D. (JPG)

S4 File Raw blots provided in support of Fig 4A–4C. (DOCX)

S5 File Raw blot provided in support of HA total extract blot in Fig 7A. (JPG)

S6 File Raw blot provided in support of EE total extract blot in Fig 7A. (JPG)

S7 File Excel sheet of raw viability data for Fig 5A. (XLSX)

S8 File Raw data provided in support of Fig 5A and S7 File. (JPG)

S9 File Raw data provided in support of Fig 6B and S10 File. (PDF)

S10 File Excel sheet with raw data and recalculated data provided in support of graph in Fig 6B. (XLSX)
