# Supplementary figures and images for: Expression of Concern: Akt Regulates Drug-Induced Cell Death through Bcl-w Downregulation
Source: PLoS One. 2019 Mar 19;14(3):e0213701. doi: 10.1371/journal.pone.0213701 (PMC6424394; doi:10.1371/journal.pone.0213701)

## Slide 1
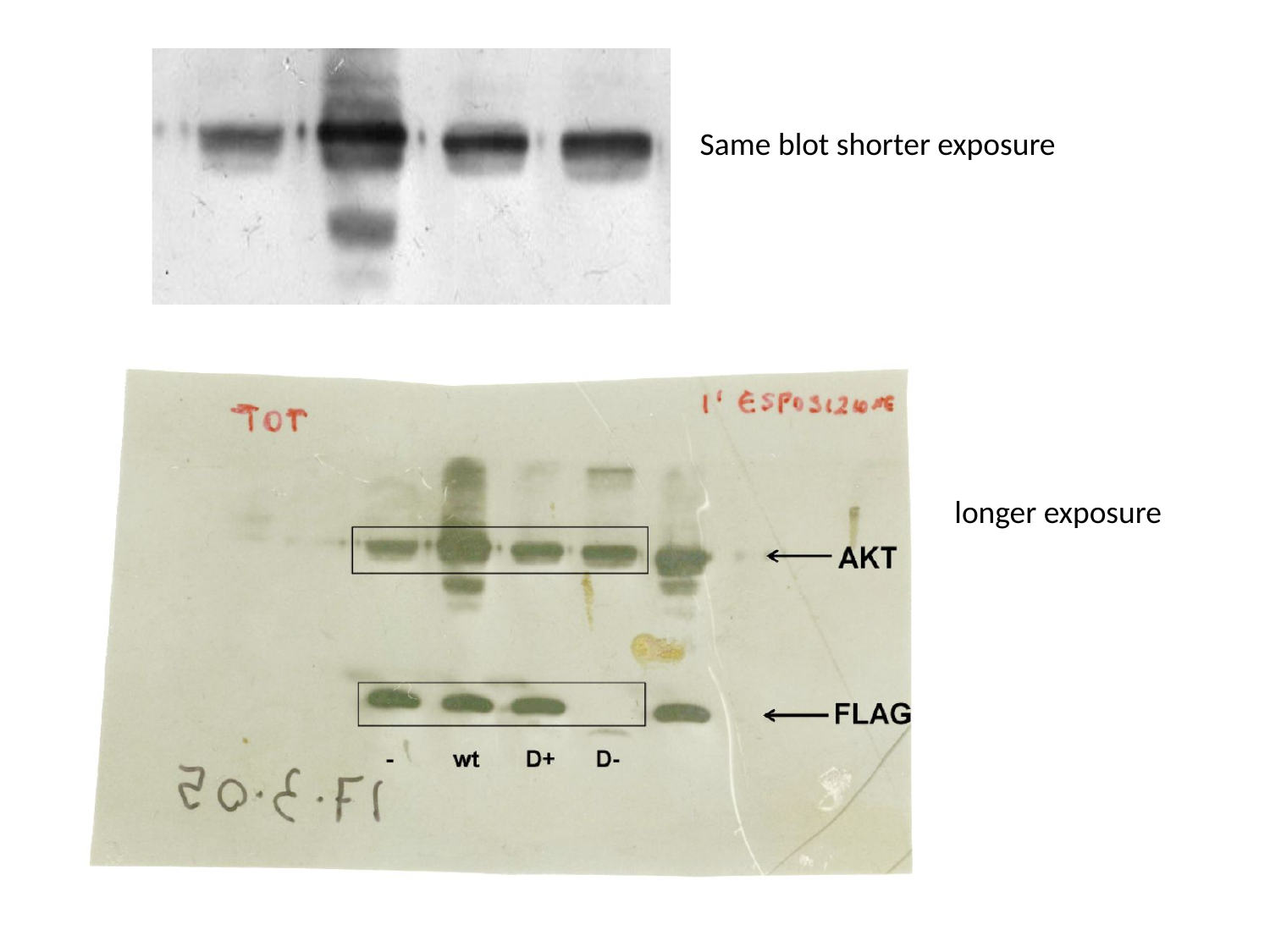

Same blot shorter exposure
longer exposure

Supplement: S1 File — (PPTX) [file pone.0213701.s001.pptx]

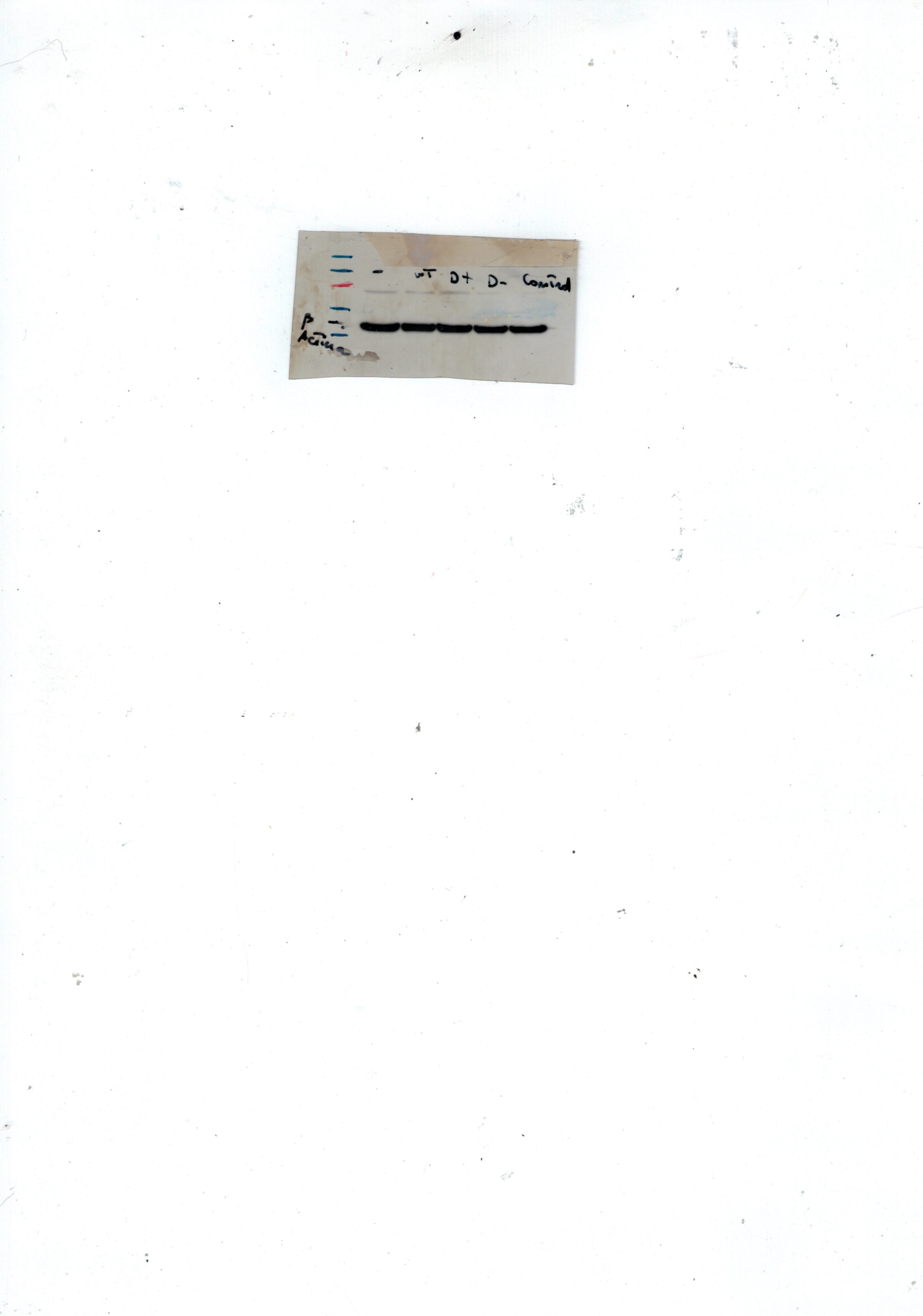

Supplement: S2 File — (JPG) [file pone.0213701.s002.jpg]

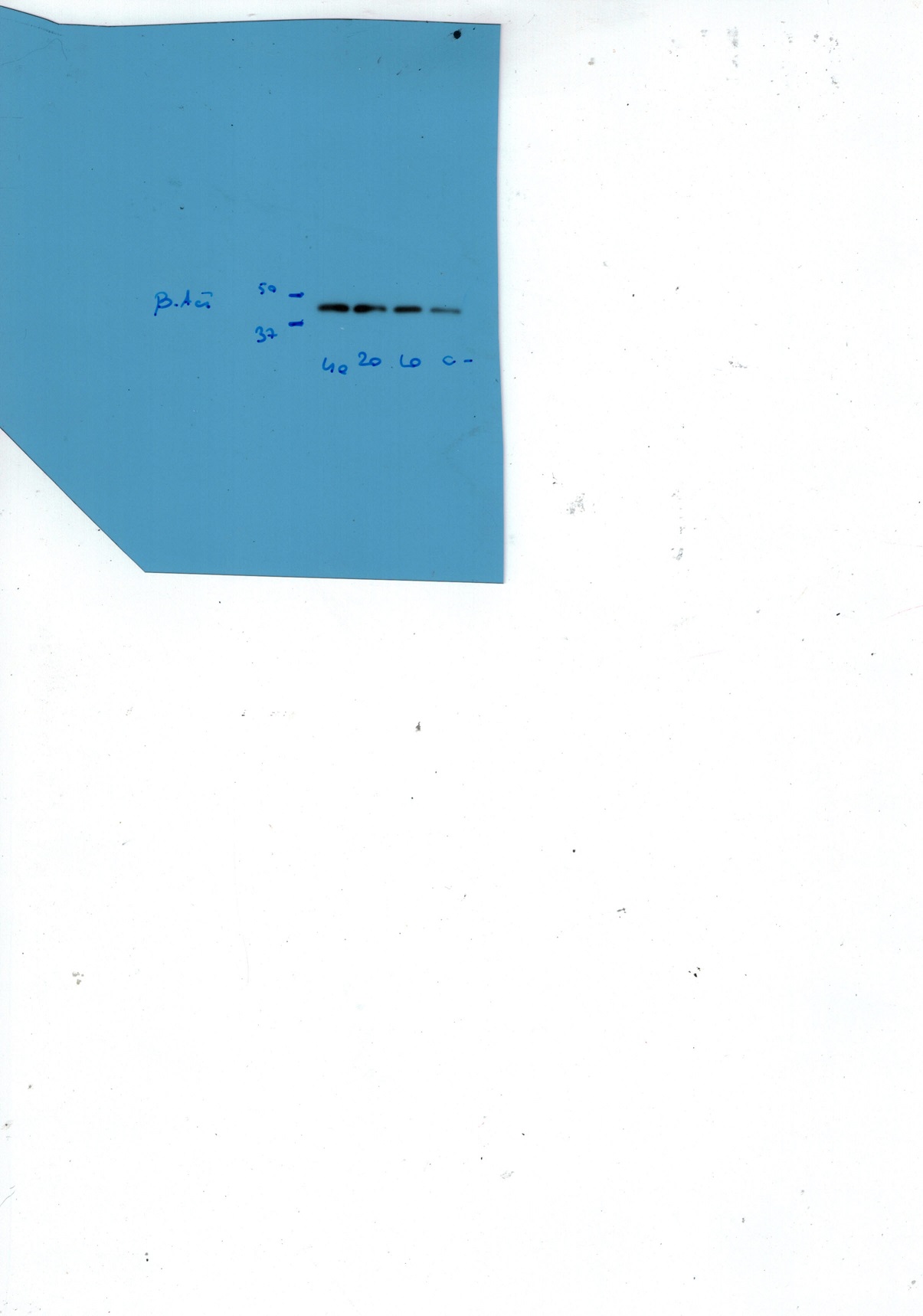

Supplement: S3 File — (JPG) [file pone.0213701.s003.jpg]

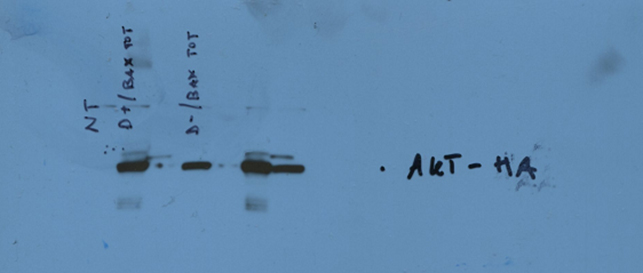

Supplement: S5 File — (JPG) [file pone.0213701.s005.jpg]

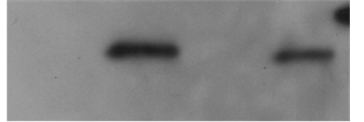

Supplement: S6 File — (JPG) [file pone.0213701.s006.jpg]

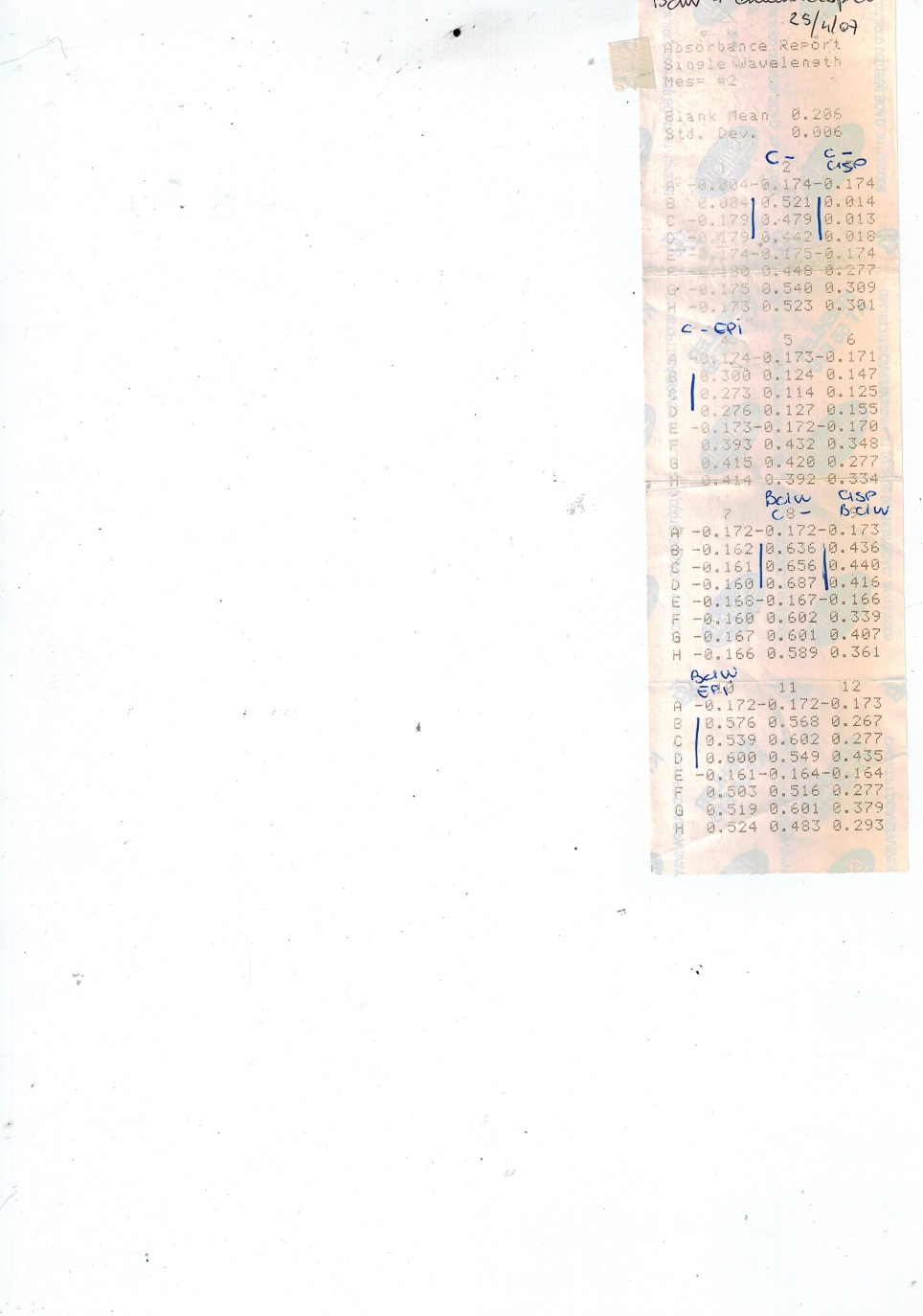

Supplement: S8 File — (JPG) [file pone.0213701.s008.jpg]
