# Supplementary material for: Expression of Concern: Akt Regulates Drug-Induced Cell Death through Bcl-w Downregulation
Source: PLoS One. 2019 Mar 19;14(3):e0213701. doi: 10.1371/journal.pone.0213701 (PMC6424394; doi:10.1371/journal.pone.0213701)

***Figure 4A: We show here the original film for the in vitro kinase assay. In the red box are indicated the bands used for the figure showed in the paper. In the figure that appears in the publication, we reduced the space between the two lanes because other samples not related to the experiment were in the middle.***

***
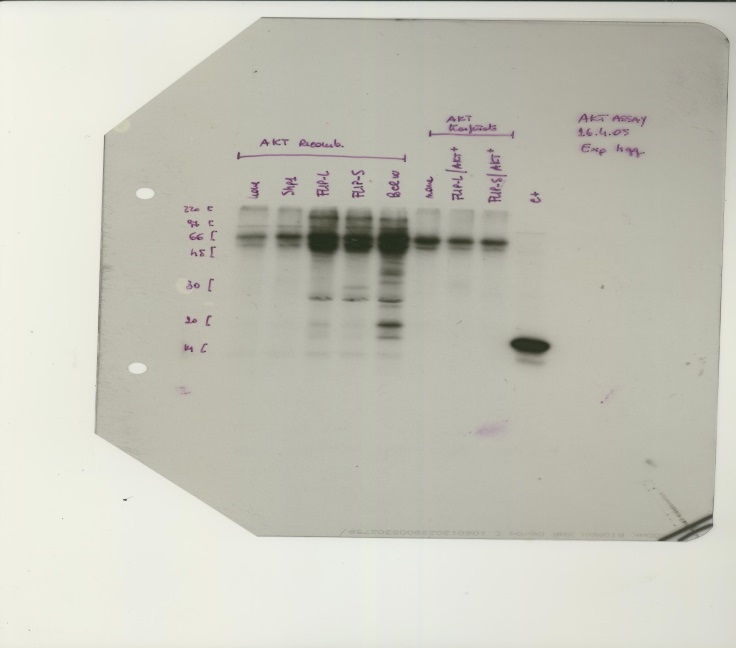
***

***Figure 4B:We show here the original films for the western blot in figure 4B***

***
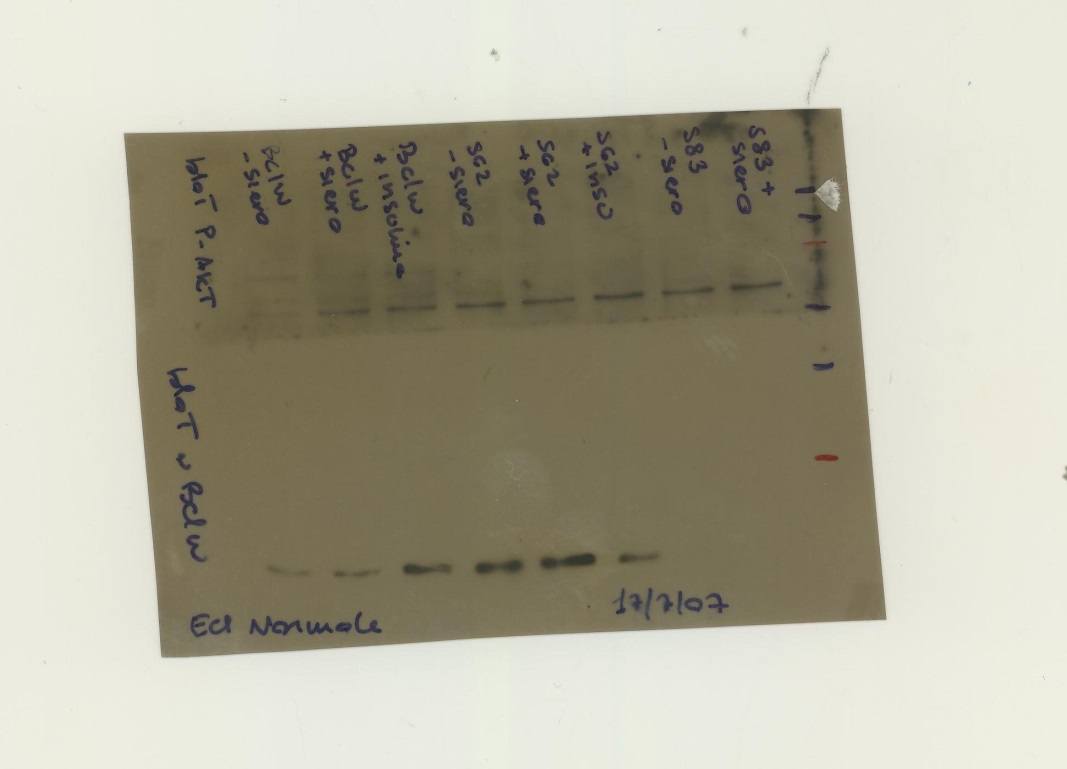

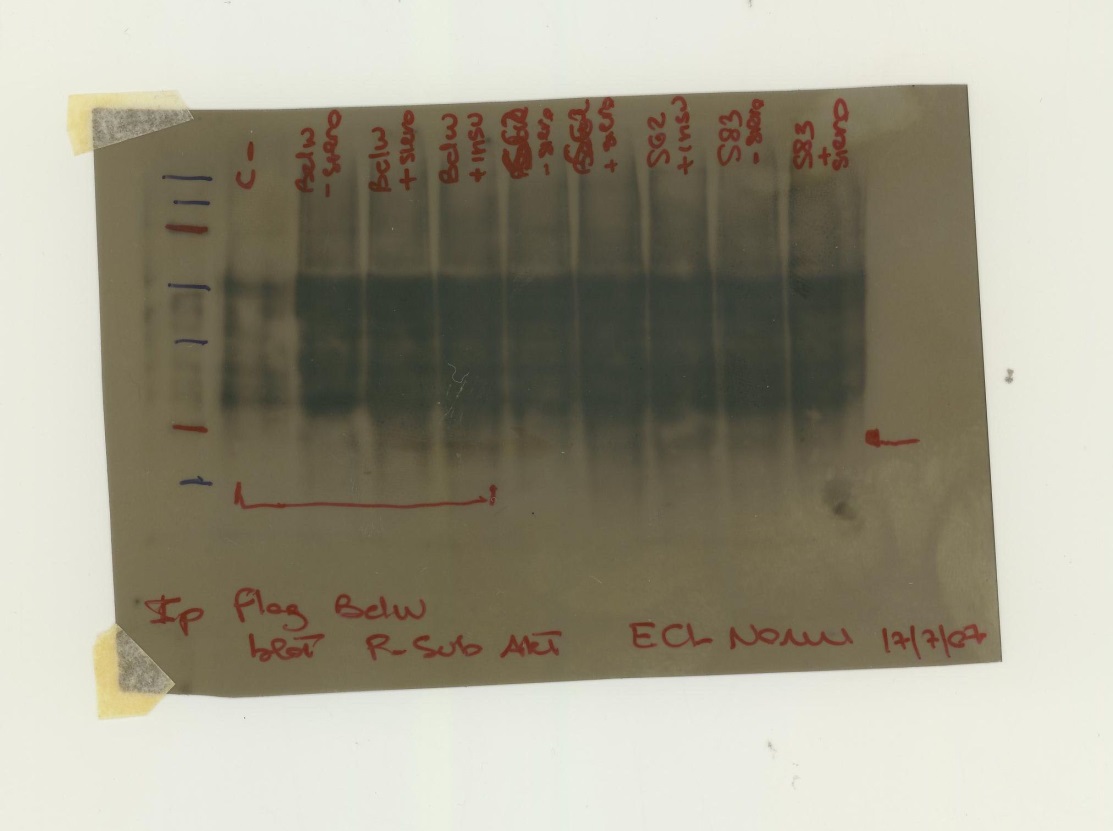
***

p-S472AKT

pSer Akt substrate

BclW

***
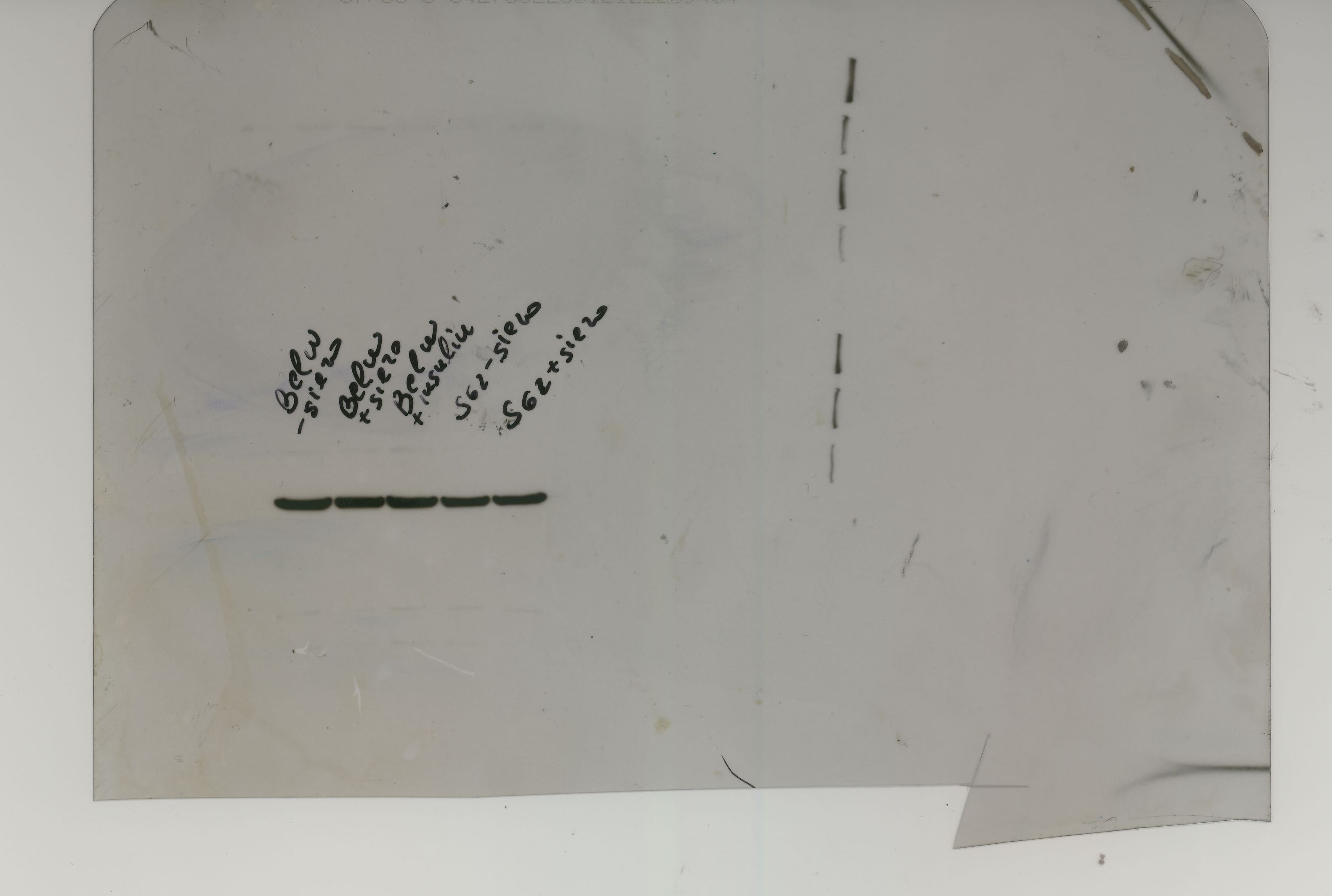
***

Actin

***The actin blot has been re-loaded to avoid errors due to stripping and re-blotting.***

***Figure 4C:***

***“We apologize for not declaring in a more clear way the splicing between the lines. Here, we show the original film used in Figure 4. We used in the published figure the lines indicated in the red box. The other lines represent another protein that we also studied, but that was not included in this paper, since not related to bclw/Akt.”***


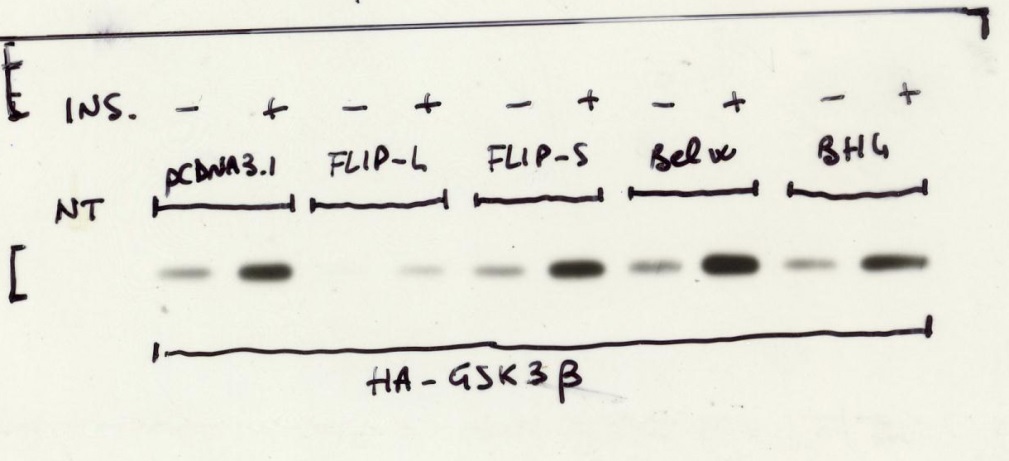


P-Gsk3β

**Figure 4 C- Flag bclw**

**This film is the same as below. Long term exposure for Flag-Bclw and lower exposure for actin (see below)**


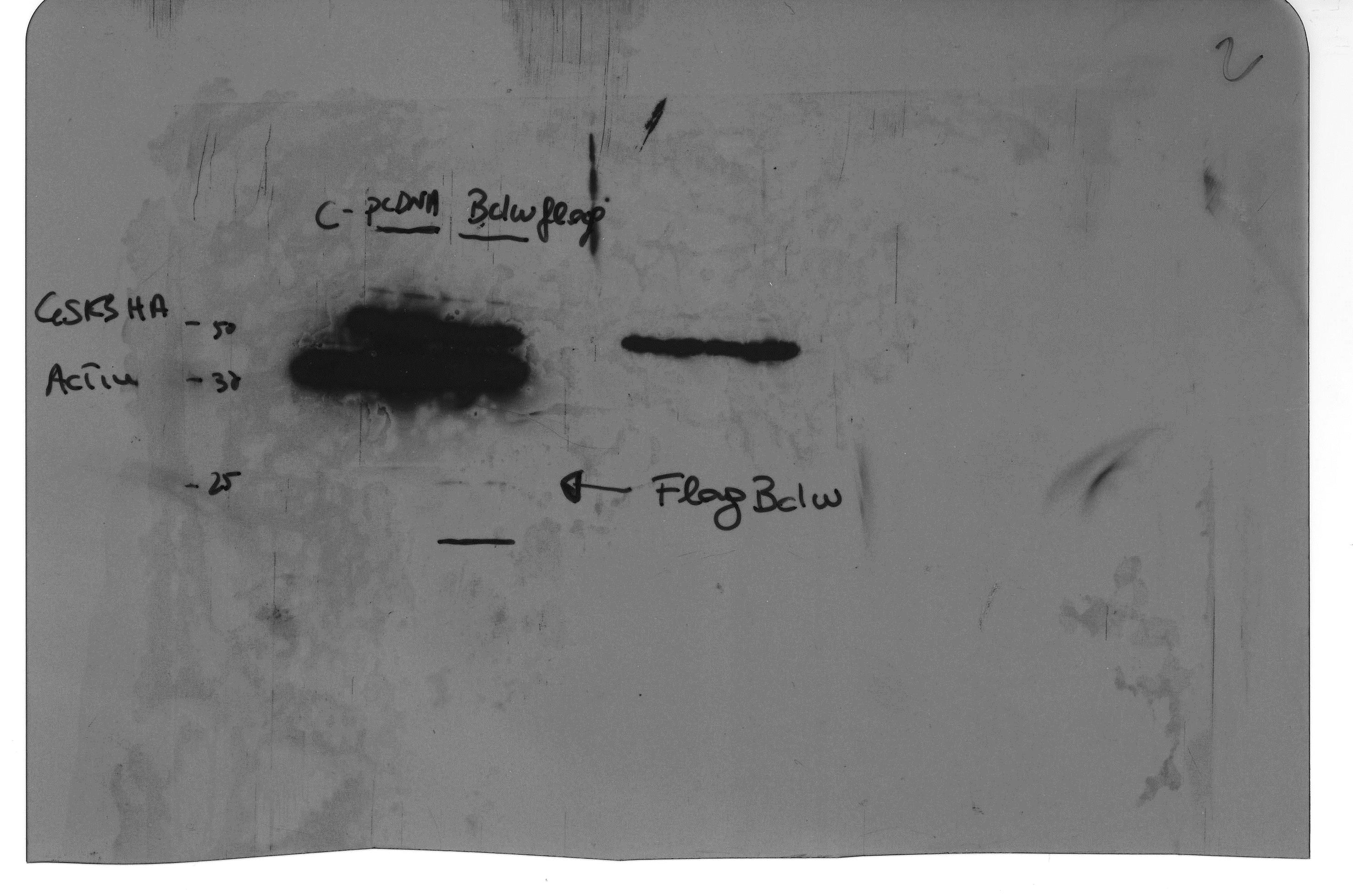


**Figure 4C actin upper panel:**

**
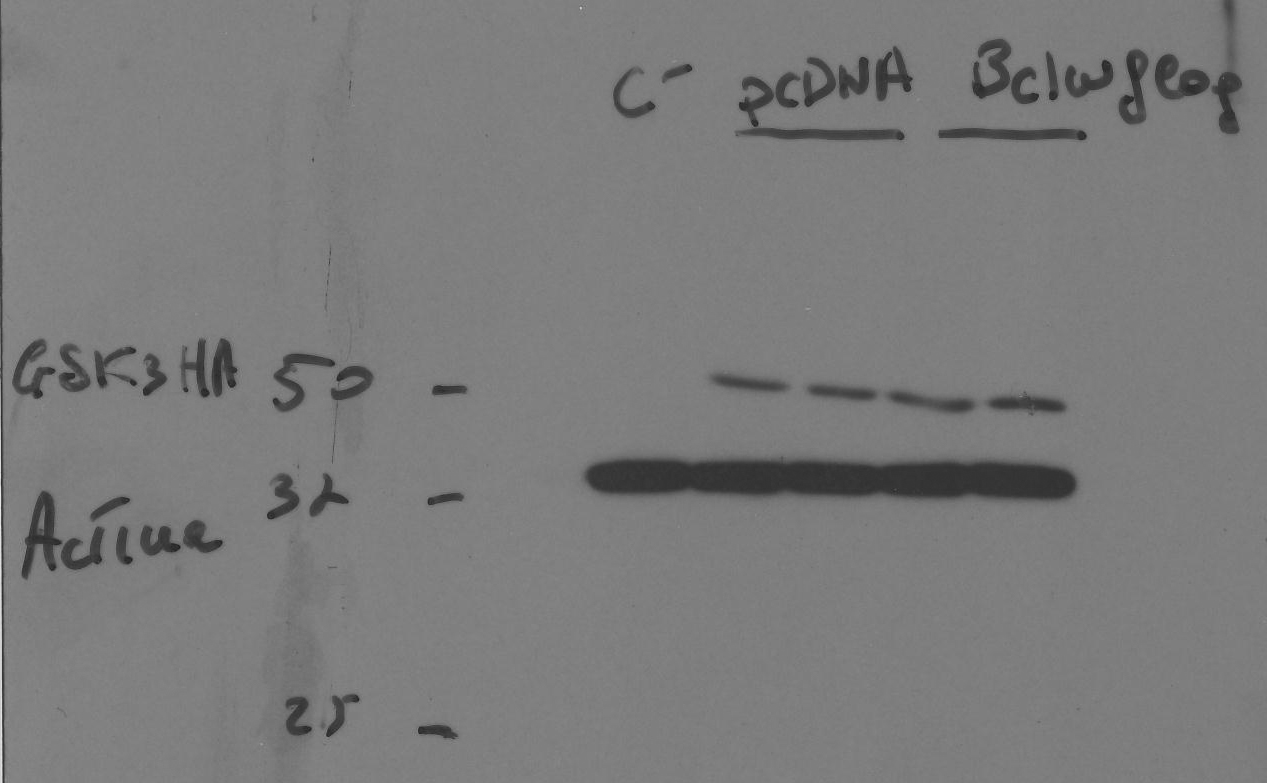
**

**Figure 4 C HA GSK3beta**

***Here we show the uncropped blot for HA-GSK3 total lysate-***


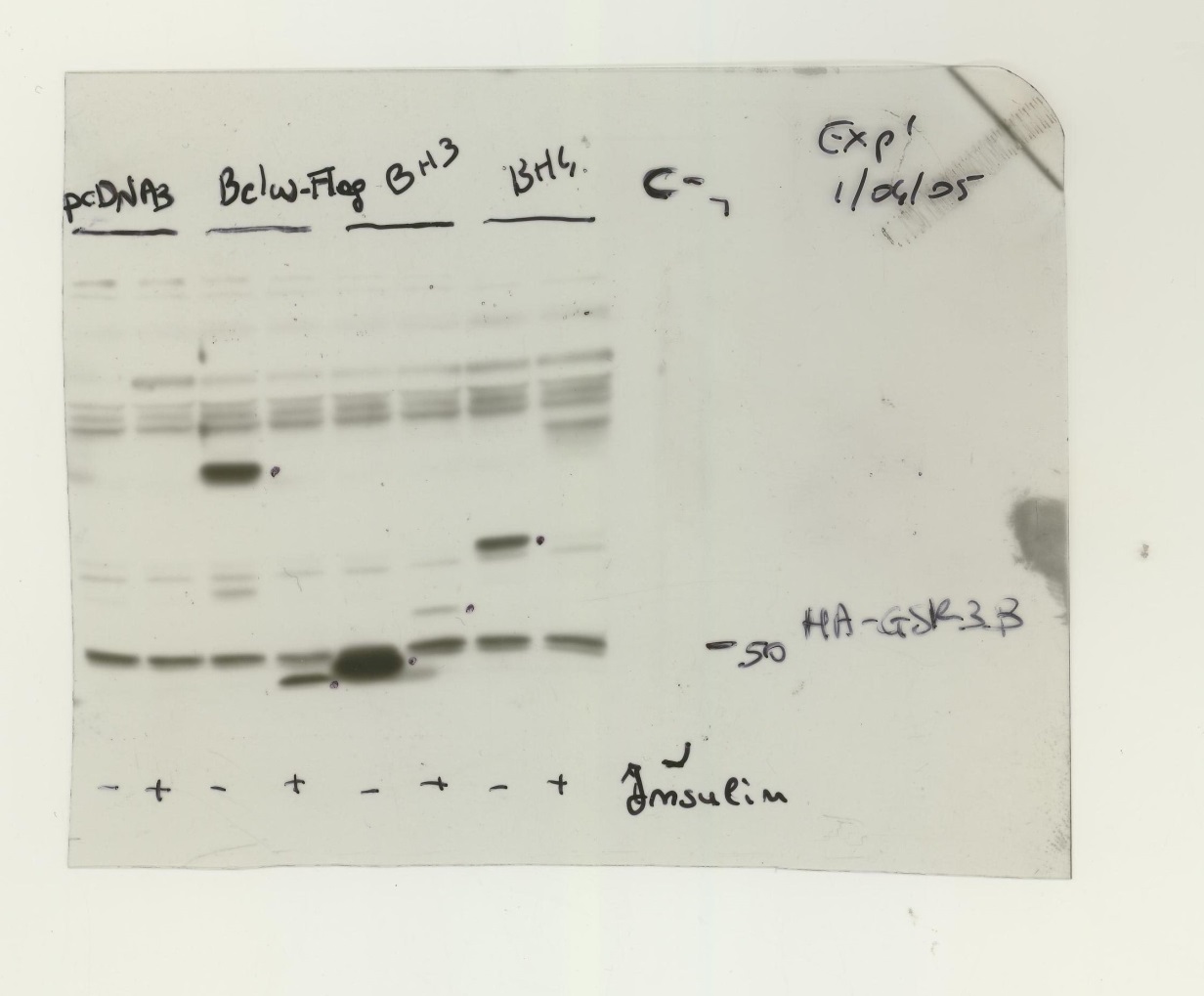


**Figure 4 C b-Actin lower panel**

***Here is the uncropped blot for β-actin for the previous panel (HA-GSK3 total lysate). The actin blot has been re-loaded to avoid errors due to stripping and re-blotting.***


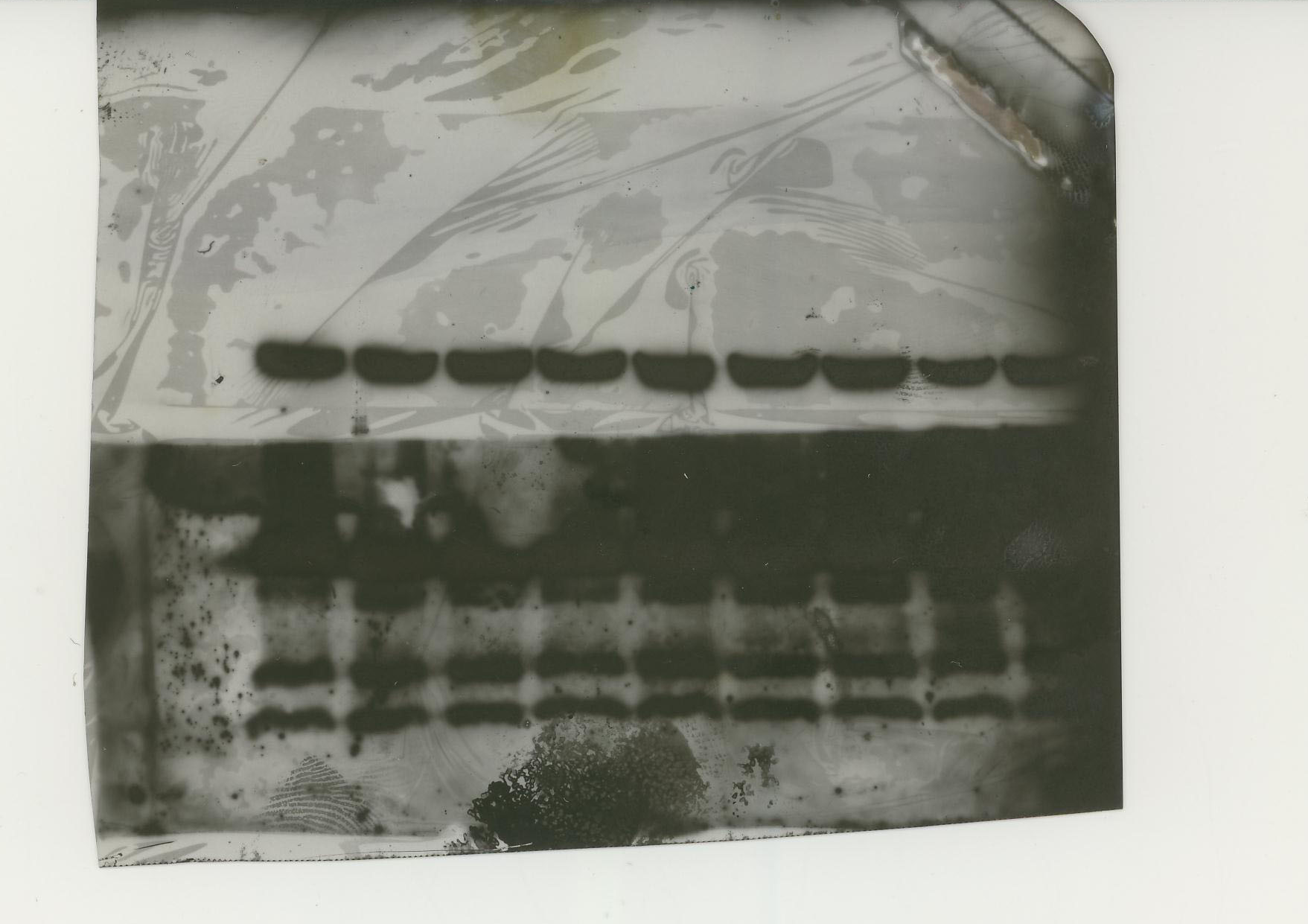

Supplement: S4 File — (DOCX) [file pone.0213701.s004.docx]
