# Supplementary material for: Expression of Concern: Akt Regulates Drug-Induced Cell Death through Bcl-w Downregulation
Source: PLoS One. 2019 Mar 19;14(3):e0213701. doi: 10.1371/journal.pone.0213701 (PMC6424394; doi:10.1371/journal.pone.0213701)
Supplement: S9 File — (PDF) [file pone.0213701.s009.pdf]

Fig 6B

Bio-Tek Instruments

Assay:A1

Wavelength:490

Date:10/03/08
Time:06:58:29AM
Temp:

Lot:
Operator:
Plate ID:

COMMENTS

Handwritten over columns 2–4, rows A/B: – GSP EPI; ~~Scrambled~~ Bclw

Handwritten over columns 6–8, rows A/B: – GSP EPI; Scrambled

CALL / CalcOD / Well / RSLT	1	2	3	4	5	6	7	8	9	10	11	12
A	0.005<br>BLK	-.132<br>SMP7	-.132<br>SMP15	-.132<br>SMP23	-.131<br>SMP31	-.130<br>SMP39	-.131<br>SMP47	-.132<br>SMP55	-.132<br>SMP63	-.132<br>SMP71	-.131<br>SMP79	-.132<br>SMP87
B	-.005<br>BLK	0.913<br>SMP8	0.507<br>SMP16	0.668<br>SMP24	-.131<br>SMP32	0.989<br>SMP40	0.557<br>SMP48	0.719<br>SMP56	-.131<br>SMP64	-.130<br>SMP72	-.131<br>SMP80	-.132<br>SMP88
C	-.131<br>SMP1	0.892<br>SMP9	0.551<br>SMP17	0.678<br>SMP25	-.131<br>SMP33	0.961<br>SMP41	0.569<br>SMP49	0.757<br>SMP57	-.132<br>SMP65	-.131<br>SMP73	-.132<br>SMP81	-.132<br>SMP89
D	-.131<br>SMP2	0.901<br>SMP10	0.543<br>SMP18	0.710<br>SMP26	-.131<br>SMP34	1.036<br>SMP42	0.616<br>SMP50	0.763<br>SMP58	-.131<br>SMP66	-.131<br>SMP74	-.131<br>SMP82	-.131<br>SMP90
E	-.131<br>SMP3	-.130<br>SMP11	-.131<br>SMP19	-.128<br>SMP27	-.130<br>SMP35	-.130<br>SMP43	-.130<br>SMP51	-.130<br>SMP59	-.131<br>SMP67	-.131<br>SMP75	-.131<br>SMP83	-.131<br>SMP91
F	-.132<br>SMP4	0.815<br>SMP12	0.213<br>SMP20	0.350<br>SMP28	-.129<br>SMP36	0.973<br>SMP44	0.377<br>SMP52	0.496<br>SMP60	-.131<br>SMP68	-.130<br>SMP76	-.131<br>SMP84	-.131<br>SMP92
G	-.127<br>SMP5	0.806<br>SMP13	0.205<br>SMP21	0.352<br>SMP29	-.130<br>SMP37	1.043<br>SMP45	0.343<br>SMP53	0.513<br>SMP61	-.131<br>SMP69	-.130<br>SMP77	-.131<br>SMP85	-.131<br>SMP93
H	-.130<br>SMP6	0.769<br>SMP14	0.200<br>SMP22	0.342<br>SMP30	-.130<br>SMP38	0.996<br>SMP46	0.352<br>SMP54	0.480<br>SMP62	-.131<br>SMP70	-.130<br>SMP78	-.131<br>SMP86	-.131<br>SMP94

Handwritten over columns 2–4, rows E/F: – GSP 61 EPI

Handwritten over columns 6–8, rows E/F: – 62 GSP EPI
